# Nintedanib promotes antitumour immunity and shows antitumour activity in combination with PD-1 blockade in mice: potential role of cancer-associated fibroblasts

**DOI:** 10.1038/s41416-020-01201-z

**Published:** 2020-12-10

**Authors:** Ryoji Kato, Koji Haratani, Hidetoshi Hayashi, Kazuko Sakai, Hitomi Sakai, Hisato Kawakami, Kaoru Tanaka, Masayuki Takeda, Kimio Yonesaka, Kazuto Nishio, Kazuhiko Nakagawa

**Affiliations:** 1 Department of Medical Oncology, Kindai University Faculty of Medicine, Osaka-Sayama, Osaka, 589-8511 Japan; 2 Department of Genome Biology, Kindai University Faculty of Medicine, Osaka-Sayama, Osaka, 589-8511 Japan

**Keywords:** Targeted therapies, Tumour immunology

## Abstract

**Background:**

Cancer-associated fibroblasts (CAFs) in the tumour microenvironment (TME) suppress antitumour immunity, and the tyrosine kinase inhibitor nintedanib has antifibrotic effects.

**Methods:**

We performed a preclinical study to evaluate whether nintedanib might enhance antitumour immunity by targeting CAFs and thereby improve the response to immune checkpoint blockade (ICB).

**Results:**

Whereas nintedanib did not suppress the growth of B16-F10 melanoma cells in vitro, it prolonged survival in a syngeneic mouse model of tumour formation by these cells, suggestive of an effect on the TME without direct cytotoxicity. Gene expression profiling indeed showed that nintedanib influenced antitumour immunity and fibrosis. Tumoural infiltration of CD8^+^ T cells and granzyme B production were increased by nintedanib, and its antitumour activity was attenuated by antibody-mediated depletion of these cells, indicating that nintedanib suppressed tumour growth in a CD8^+^ T cell-dependent manner. Moreover, nintedanib inhibited the proliferation and activation of fibroblasts. Finally, the combination of nintedanib with ICB showed enhanced antitumour efficacy in B16-F10 tumour-bearing mice.

**Conclusions:**

Our results suggest that nintedanib targeted CAFs and thereby attenuated the immunosuppressive nature of the TME and promoted the intratumoural accumulation and activation of CD8^+^ T cells, with these effects contributing to enhanced antitumour activity in combination with ICB.

## Background

The development of immune checkpoint blockade (ICB) over the last decade has led to a major shift in cancer treatment for individuals with a wide range of tumour types. ICB therapy, which targets receptor–ligand interactions of molecules such as cytotoxic T lymphocyte-associated protein-4 (CTLA-4) and programmed cell death-1 (PD-1), thus provides substantial clinical benefit in terms of a durable response in some patients. However, most patients fail to respond to ICB,^1^ with resistance mechanisms being associated with tumour immunogenicity, antigen presentation, oncogenic signalling pathways and the tumour microenvironment (TME).^1^ Characterisation of the TME is therefore important to provide insight into mechanisms of ICB resistance and for the development of effective combination strategies to overcome such resistance.

The TME (also referred to as the tumour stroma) comprises all the noncancer cell components of a tumour, including fibroblasts, myeloid-derived suppressor cells, macrophages, lymphocytes, extracellular matrix (ECM) and intertwined blood vessels formed by endothelial cells and pericytes.^2–4^ Such cancer-associated stromal cells, together with inhibitory cytokines in the TME, give rise to an immunosuppressive niche in which tumour cells are protected from antitumour immune cells and thereby promote failure of ICB therapy.^1^ Targeting of the TME is thus a promising approach to increase the efficacy of ICB.^1^ Indeed, inhibition of vascular endothelial growth factor (VEGF), which acts on endothelial cells to stimulate angiogenesis and limits immune cell activity, was found to be associated with an improved response to ICB therapy in renal cell carcinoma.^5^

Fibroblasts in normal tissue are generally quiescent. However, these cells become activated during cancer development, tumour-promoting inflammation, and tumour fibrosis.^6^ Such activated fibroblasts found in association with cancer are termed cancer-associated fibroblasts (CAFs), although they are also known as tumour-associated fibroblasts, myofibroblasts, or reactive stromal fibroblasts.^7^ Among the cellular components of the TME, CAFs are the most abundant and play a key role in cancer progression.^4^ They contribute to the establishment of an immunosuppressive TME by influencing antitumour immune cells. Deposition of ECM components—in particular, fibrillar collagen and fibronectin—by CAFs in the tumour stroma can result in the formation of a physical barrier to immune cell infiltration.^4,8^ In addition, CAFs release large amounts of growth factors and proinflammatory cytokines that recruit immunosuppressive cells into the tumour stroma and thereby promote immune evasion.^4,9^

Nintedanib (BIBF1120) is an oral small-molecule inhibitor of receptor tyrosine kinases including fibroblast growth factor receptor (FGFR) 1 to 3, platelet-derived growth factor receptor (PDGFR) α and β, and VEGF receptor (VEGFR) 1 to 3 and has been approved for the treatment of lung adenocarcinoma as well as idiopathic pulmonary fibrosis.^10,11^ In vitro and in vivo experiments have shown that nintedanib inhibits several steps in the initiation and progression of lung fibrosis, including the release of proinflammatory and profibrotic mediators, the migration and differentiation of fibrocytes and fibroblasts, and the deposition of ECM.^12–14^ The antifibrotic activity of nintedanib and immunosuppressive role of CAFs suggest that nintedanib might attenuate CAF activation and enhance antitumour immunity by inhibiting CAF-mediated immunosuppression, with these effects possibly giving rise to synergistic antitumour activity in combination with ICB. We therefore performed a preclinical study to evaluate whether nintedanib is able to abrogate the immunosuppression associated with the TME by targeting CAFs and thereby to improve the tumour response to ICB.

## Methods

### Cell lines and reagents

B16-F10, NIH-3T3, A549 and H1703 cells were obtained from American Type Culture Collection. Cells were maintained under a humidified atmosphere of 5% CO_2_ at 37 °C in Dulbecco’s modified Eagle’s medium (Sigma–Aldrich) for B16-F10 and NIH-3T3 or in RPMI-1640 medium (Sigma–Aldrich) for A549 and H1703. Each medium was supplemented with 10% heat-inactivated foetal bovine serum (FBS) (Biowest) and 1% penicillin–streptomycin–amphotericin B (Wako). Nintedanib was obtained from ChemScene. Antibodies to PD-1 (clone RMP1-14) and to CD8 (clone 53–6.7) for in vivo administration were obtained from Bio X Cell. For examination of the antifibrotic activity of nintedanib, NIH-3T3 mouse fibroblasts were stimulated with recombinant transforming growth factor–β1 (TGF-β1) (R&D Systems) at 5 ng/ml in the presence of various concentrations of nintedanib either for 48 h for immunoblot analysis or for 72 h for in vitro assay of cell viability.^15–17^

### Immunoblot analysis

Immunoblot analysis was performed as previously described.^18^ Cells were seeded at a density of 1.2 × 10^6^/well in six‑well plates, cultured for 24 h, harvested by exposure to trypsin, washed with ice-cold phosphate-buffered saline (PBS), and lysed in a solution containing 25 mM Tris-HCl (pH 8.3), 192 mM glycine, 0.1% SDS, and 1 mM phenylmethylsulphonyl fluoride. Tumour or normal tissue was homogenised in the same lysis solution. Cell and tissue lysates were centrifuged at 12,000 × *g* for 10 min at 4 °C, and the resulting supernatant was assayed for protein content with a BCA Protein Assay Kit (Pierce). Portions (35 μg of protein) of each sample were subjected to SDS-polyacrylamide gel electrophoresis (PAGE) on a 7.5% gel, and the separated proteins were transferred to a nitrocellulose membrane. The membrane was then incubated overnight at 4 °C with antibodies to VEGFR-2 (1:1000 dilution; catalog no. 9698, Cell Signaling Technology), to PDGFR-α (1:1000; 3164, Cell Signaling Technology), to FGFR-2 (1:2000; ab10648, Abcam), to FAP (1:1000; ab28244, Abcam) or to β‑actin (1:100; A2066, Sigma–Aldrich). Immune complexes were detected with horseradish peroxidase–conjugated secondary antibodies (1:10.000; NA934V, GE Healthcare) and enhanced chemiluminescence reagents (GE Healthcare). Immunoblots were scanned with an Amersham Imager 680 (GE Healthcare), and protein band intensities were quantified with the use of Image J software (http://rsb.info.nih.gov/ij) and normalised by the loading control.

### Cell viability and proliferation assays

For assay of cell viability, B16-F10, H1703, A549 and NIH-3T3 cells were plated in 96-well round-bottomed plates at a density of 2 × 10^3^/well and incubated for 72 h with various concentrations of nintedanib. Cell viability was then assessed with the use of a CellTiter-Glo 3D Luminescent Cell Viability Assay (Promega). For assay of cell proliferation, B16-F10 or NIH-3T3 cells were seeded in 10-cm dishes and exposed to 0.5 or 1 μM nintedanib for 24, 48 or 72 h. The cells were then washed three times with ice-cold PBS, collected with the use of Accutase solution (BD Biosciences), and stained with the use of a Zombie Fixable Viability Kit (BioLegend) for discrimination of live from dead cells. The living cells were counted with a flow cytometer (LSRFortessa X-20, BD Biosciences) and normalised by those in untreated cultures.

### In vivo experiments

All mice were housed under specific pathogen-free conditions. Six-week-old female C57BL/6 mice (CLEA Japan) with a body weight of 16–20 g were injected subcutaneously with 5 × 10^5^ B16-F10 cells on the right flank. When tumour volume had reached 30–70 mm^3^, mice were randomly assigned to treatment arms. For analysis of tumour growth and survival, mice received nintedanib (50 mg/kg) or vehicle orally five times a week. For examination of the effects of combination treatment, mice received an antibody to PD-1 (10 mg/kg) intraperitoneally twice a week or a combination of nintedanib and anti–PD-1. Nintedanib was suspended in 0.5% methylcellulose by ultrasonic treatment and was administered intragastrically with a gavage needle. All treatment regimens were continued for up to 2 weeks. For in vivo depletion of CD8^+^ cells, an antibody to CD8 (10 mg/kg) was administered intraperitoneally 2 days before and on the day of the first dose of nintedanib and then weekly until the end of the experiment. Tumour volume and body weight were measured twice per week. Tumour volume was calculated as: 0.5 × length × width^2^. Mice were killed by cervical dislocation when tumours became necrotic, grew to a volume of 1200 mm^3^, or were harvested for analysis.

### Microarray analysis

Microarray analysis was performed as described previously.^19^ Total RNA was isolated from nintedanib- or vehicle-treated B16-F10 tumours with the use of a RNeasy Mini Kit (Qiagen). RNA yield and integrity were assessed with a NanoDrop 2000 spectrophotometer (Thermo Scientific) and an Agilent Bioanalyzer (Agilent Technologies), respectively. The analysis was conducted with a GeneChip Mouse Gene 2.0 ST Array (Affymetrix), and data processing and normalisation were performed with Affymetrix Transcriptome Analysis Console (TAC) software (v.4.0). Biological interpretation of gene expression profiles was conducted with the use of DAVID bioinformatics resources (https://david-d.ncifcrf.gov), Gene Set Enrichment Analysis (GSEA) 4.0 software, and Ingenuity Pathway Analysis (IPA) software.

### Immunohistochemical analysis and Sirius red staining

Immunohistochemical analysis was performed as previously described.^20^ In brief, sections (thickness of 5 µm) of formalin-fixed, paraffin-embedded tumour tissue were placed in a heat chamber at 60 °C for 15 min to remove paraffin and then subjected to blocking of endogenous peroxidase activity followed by heat-mediated antigen retrieval. After blocking of nonspecific sites with 5% goat serum and 2.5% bovine serum albumin, the sections were incubated for 30–60 min at room temperature or overnight at 4 °C with antibodies to CD8 (1:1000 dilution; clone D4W2Z; catalog no. 98941, Cell Signaling Technology), to CD4 (1:1000; EPR19514; ab183685, Abcam), to granzyme B (1:100; E5V2L; 44153, Cell Signaling Technology), to α-SMA (1:500; 1A4; M0851, Dako) or to CD31 (1:300; SZ31; DIA-310, Dianova). Immune complexes were subsequently detected with an anti-rabbit or anti-mouse Histofine Simple Stain MAX PO horseradish peroxidase–conjugated polymer (Nichirei Biosciences) and the peroxidase substrate AEC (Vector Laboratories). Sections were counterstained with haematoxylin. For Sirius red staining of collagen, sections were exposed to Direct Red 80 (Sigma–Aldrich). All slides were examined with a BZ-X700 microscope (Keyence) or a Nanozoomer scanner (Hamamatsu). For quantification of CD8^+^ or CD4^+^ tumour-infiltrating lymphocytes (TILs), the intra- and peritumoural borders were identified by haematoxylin-eosin staining and observation at low magnification (×4 objective lens). Five nonoverlapping fields were then randomly selected for each of the intratumoural and peritumoural regions, the slides were examined at high magnification (×20 objective lens), the number of TILs was counted with the use of Image J software, and the average number of TILs was determined for both intratumoural and peritumoural tissue. For quantification of granzyme B and α-SMA immunoreactivity as well as collagen staining, five nonoverlapping fields were randomly selected, the slides were examined at high magnification (×20 objective lens), the positive area (%) was measured after setting up a threshold with the use of Image J software, and the average positive area was determined. For quantification of CD31 immunoreactivity, the number of microvessel structures was determined in a manner similar to that for quantification of CD8^+^ and CD4^+^ TILs.

### ELISA for FGF-2

The concentration of fibroblast growth factor–2 (FGF-2) was measured with the use of a mouse FGF enzyme-linked immunosorbent assay (ELISA) kit (R&D Systems). Cells were plated at a density of 1.2 × 10^6^/well in six-well plates, cultured for 24 h, and then incubated in serum-free medium for an additional 24 h, after which the medium was collected for assay of FGF-2 and the cells were lysed in Cell Lysis Buffer 2 (R&D Systems). Tumour and normal tissue resected from mice was homogenised in 300 μl of PBS and 300 μl of Cell Lysis Buffer 2. Both cell and tissue lysates were centrifuged at 12,000 × *g* for 10 min at 4 °C, and the resulting supernatants were assayed for FGF-2. The amount of FGF-2 was normalised by the amount of total input protein.

### Flow cytometric analysis

Flow cytometric analysis was performed as previously described.^21^ Tumour or normal skin tissue resected from mice was mechanically dissociated and digested with the use of a gentleMACS system (Miltenyi) and by exposure to collagenase type IV (200 U/ml, Sigma–Aldrich) and DNase I (100 μg/ml, Sigma–Aldrich), and a single-cell suspension was obtained by passage of the dissociated and digested tissue through a 70-μm cell strainer. The cells were washed three times with ice-cold PBS and then exposed to Fc block (2.4G2, BD Pharmingen). In the case of cultured cells, the cells were dissociated with the use of Accutase solution (BD Biosciences). The tissue-derived and cultured cells were then stained first with the use of a Zombie Fixable Viability Kit (BioLegend) for discrimination of live from dead cells and then with antibodies to surface marker proteins. They were washed twice with Stain Buffer containing FBS (BD Biosciences) before flow cytometry. Antibodies to the following proteins were used: CD45 (clone 30-F11; catalog no. 103149, BioLegend), CD31 (390; 102423, BioLegend) and FAP (ab28244, Abcam). Goat secondary antibodies to rabbit immunoglobulin G (A-10931, Invitrogen) were used for detection of mouse FAP. All antibodies were diluted with Stain Buffer containing FBS. Flow cytometry was performed with an LSRFortessa X-20 instrument (BD Biosciences), and the data were analysed with FlowJo software. Fluorescence minus one (FMO) controls and corresponding isotype controls were included for each analysis. See Supplementary Fig. 1 for the gating strategy.

### Statistical analysis

Data are presented as means ± SEM unless indicated otherwise. Continuous variables were compared between two groups with the unpaired *t*-test or among more than two groups by one-way analysis of variance (ANOVA) with Tukey’s correction for multiple comparisons. Differences in survival curves constructed by the Kaplan–Meier method were assessed with the log-rank test. Missing data were not imputed. All statistical analysis was performed with JMP software version 10.0.2 (SAS Institute). Data were graphically depicted with GraphPad Prism 8.3 (GraphPad Software). *P* values were based on a two-sided hypothesis, and those of <0.05 were considered statistically significant.

## Results

### Nintedanib manifests antitumour efficacy without direct cytotoxicity

**Fig. 1:**
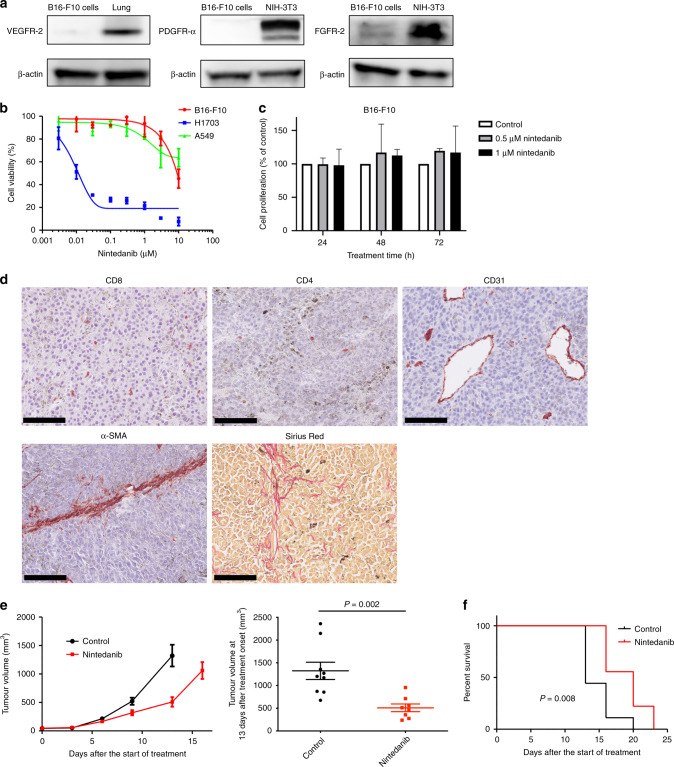
Nintedanib shows antitumour efficacy without direct cytotoxicity. **a** Immunoblot analysis of VEGFR-2, PDGFR-α, FGFR-2 and β-actin (loading control) in B16-F10 cells. Normal mouse lung tissue served as a positive control for VEGFR-2 expression, as did NIH-3T3 cells for PDGFR-α and FGFR-2 expression. **b** Cell viability assay for nintedanib and either B16-F10, H1703 (positive control) or A549 (negative control) cells. Data are means ± SEM for two independent experiments, each performed with six technical replicates. **c** Cell proliferation assay for nintedanib and B16-F10 cells. Cells were treated with 0.5 or 1 μM nintedanib for 24, 48 or 72 h, after which living cells were counted by flow cytometry and normalised by those in untreated samples. Data are means + SEM for two independent experiments, each performed with two technical replicates. No significant differences were apparent between untreated and treated samples at the same treatment time (one-way ANOVA with Tukey’s correction for multiple comparisons). **d** Representative immunohistochemical staining of CD8 and CD4 for T cells, CD31 for microvessels and α-SMA for CAFs as well as representative Sirius red staining of collagen in B16-F10 tumours derived from mice. Scale bars, 100 μm. **e** Time course of tumour volume (left) as well as tumour volume at 13 days after treatment initiation (right) for subcutaneous B16-F10 tumours treated with nintedanib or vehicle (control). Data are means ± SEM for seven or eight tumours in each group. The *P* value was determined with the unpaired *t*-test. **f** Survival curves for the B16-F10 tumour-bearing mice in **e**. The *P* value was determined with the log-rank test.

We first examined expression of the key molecular targets of nintedanib in B16-F10 mouse melanoma cells. Immunoblot analysis revealed that VEGFR-2, PDGFR-α and FGFR-2 were essentially undetectable in these cells (Fig. 1a). We next evaluated the antitumour efficacy of nintedanib in vitro and found that it did not attenuate the viability of B16-F10 cells or that of A549 human lung adenocarcinoma cells (examined as a negative control) at concentrations up to 1 µM (Fig. 1b).^22^ Nintedanib did attenuate the viability of H1703 human lung squamous cell carcinoma cells (Fig. 1b), which served as a positive control on the basis of the previous detection of *FGFR1* and *PDGFRA* amplification in these cells^22,23^ and of our finding that they express PDGFR-α and FGFR-2 (Supplementary Fig. 2). We also found that nintedanib did not inhibit the proliferation of B16-F10 cells (Fig. 1c). These results thus indicated that nintedanib does not show direct cytotoxicity for B16-F10 cells.

Given that regulation of the TME by nintedanib might be expected to have an antitumour effect,^13^ we performed in vivo experiments with a syngeneic mouse tumour model established by subcutaneous injection of B16-F10 cells. We first examined B16-F10 tumour tissue resected from mice for TME components including infiltrating immune cells, vasculature, CAFs, and collagen produced by fibroblastic cells. Histopathologic analysis indeed revealed the presence in B16-F10 tumours of CD8^+^ and CD4^+^ T cells, CD31^+^ microvessel structures, α–smooth muscle actin (α-SMA)–positive CAFs, and collagen (stained with Sirius red) (Fig. 1d). We further found that nintedanib treatment did not influence the number of CD31^+^ microvessel structures in this model (Supplementary Fig. 3), although it has been shown to possess marked antiangiogenic activity.^22^ Nintedanib treatment resulted in a marked delay in tumour growth (Fig. 1e) and significantly prolonged the survival of the model mice (Fig. 1f) compared with vehicle treatment. No loss of body weight was observed during the course of nintedanib treatment, suggestive of a lack of health-related toxicity. Given that nintedanib did not show a direct antiproliferative effect on B16-F10 cells in vitro, its antitumour efficacy in vivo might thus be mediated by effects on cellular components of the tumour stromal compartment such as immune cells or CAFs.

### Gene expression profiling reveals effects of nintedanib on antitumour immunity and fibrosis

**Fig. 2:**
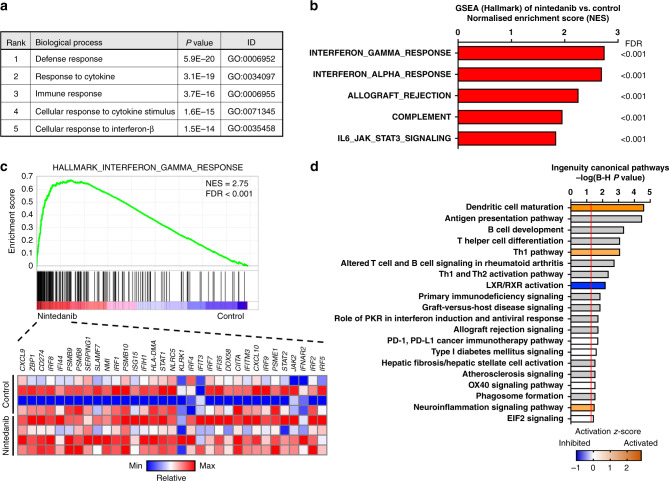
Gene expression profiling reveals that nintedanib influences antitumour immunity and fibrosis. **a** Gene ontology (GO) analysis by DAVID of upregulated genes (>1.5-fold change in expression) in nintedanib-treated versus vehicle-treated (control) B16-F10 tumours (*n* = 4 for each) as determined by microarray analysis. The top five significantly enriched biological processes are listed. The *P* values were determined by Fisher’s exact test, with the Bonferroni correction being applied for multiple comparisons. **b** GSEA of upregulated gene signatures in nintedanib-treated versus control tumours (*n* = 4 for each) as determined by microarray analysis. The top five gene signatures from the Hallmark collection of MSigDB are listed. FDR, false discovery rate. **c** GSEA plot of enrichment for the gene signature related to the interferon-γ response from MSigDB for nintedanib-treated versus control tumours (top) as well as a heat map for genes in this signature for each of the four tumour replicates in each group (bottom). **d** Top 20 significant canonical pathways identified by IPA for nintedanib-treated versus control tumours. Orange and blue bars represent activated and inhibited pathways, respectively, whereas grey bars indicate no overall change in pathway activity but a highly significant association of individual genes in the pathway. *P* values were corrected for multiple testing with the Benjamini–Hochberg (B–H) method, with the vertical red line indicating a B–H–corrected *P* value of 0.05.

To evaluate the effects of nintedanib on the TME, we first performed gene expression profiling by microarray analysis for B16-F10 tumours treated with vehicle or nintedanib for 7 days. Differential expression analysis revealed that the expression of 175 genes was upregulated (>1.5-fold change) and that of 57 genes was down-regulated (<0.67-fold change) in nintedanib-treated tumours compared with vehicle-treated tumours. An unbiased DAVID gene ontology (GO) analysis of upregulated genes in nintedanib-treated tumours relative to control tumours showed that the top 20 biological processes included categories related to immune responses and responses to interferon (Fig. 2a; Supplementary Fig. 4). GSEA revealed that, whereas highly upregulated gene signatures in nintedanib-treated tumours included those related to interferon responses (Fig. 2b, c), gene signatures related to fibroblasts were highly down-regulated in nintedanib-treated tumours (Supplementary Fig. 5). IPA for canonical pathways showed that genes whose expression was altered in nintedanib-treated tumours were significantly associated with pathways related to immunity and fibrosis (Fig. 2d). Collectively, these findings suggested that nintedanib influenced antitumour immunity and stromal fibroblasts in the TME.

### Nintedanib promotes antitumour immunity in B16-F10 tumours

**Fig. 3:**
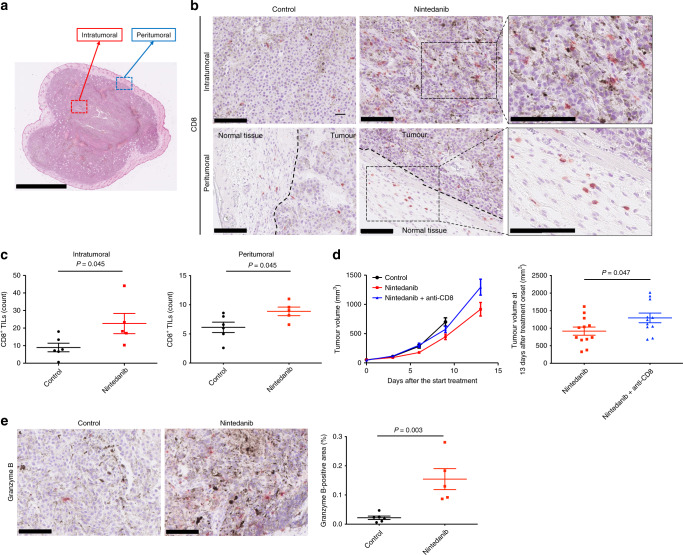
Nintedanib promotes antitumour immunity in B16-F10 tumours. **a** Representative haematoxylin-eosin–stained section of a B16-F10 tumour indicating the intra- and peritumoural borders. Scale bar, 2.5 mm. **b** Representative immunohistochemical staining for CD8 in intra- and peritumoural regions of B16-F10 tumours derived from mice treated with vehicle or nintedanib for 7 days. Scale bars, 100 μm. **c** Number of CD8^+^ TILs in intra- and peritumoural regions of B16-F10 tumours determined from sections as in **b**. Data are means ± SEM for five or six mice per group. The *P* values were determined with the unpaired *t*-test. **d** Time course of tumour volume (left) as well as tumour volume at 13 days after treatment onset (right) for B16-F10 tumours treated with vehicle or with nintedanib either alone or together with an antibody to CD8 to deplete CD8^+^ cells. Data are means ± SEM for 11 or 12 tumours in each arm (pooled from two independent experiments). The *P* value was determined with the unpaired *t*-test. **e** Representative immunohistochemical staining (left) as well as the percentage positive area (right) for granzyme B in B16-F10 tumours derived from mice treated with vehicle or nintedanib for 7 days. Scale bars, 100 μm. Quantitative data are means ± SEM for five or six mice per group. The *P* value was determined with the unpaired *t*-test.

To address further the notion that nintedanib influences the TME, we next focused on CD8^+^ T cells, which play a key role in the TME. We thus counted the number of CD8^+^ lymphocytes in both intratumoural and peritumoural tissue of B16-F10 tumours by immunohistochemical analysis (Fig. 3a, b). The number of CD8^+^ cells in both intratumoural and peritumoural regions was significantly higher for nintedanib-treated tumours than for vehicle-treated tumours (Fig. 3c). To evaluate whether such CD8^+^ T cells are required for the observed antitumour effect of nintedanib, we treated B16-F10 tumour-bearing mice with an antibody to CD8. Depletion of CD8^+^ T cells indeed impaired the antitumour efficacy of nintedanib (Fig. 3d). We also examined immunoreactivity for granzyme B, a marker of T cell activation, and detected an increase in granzyme B production in nintedanib-treated tumours compared with vehicle-treated tumours (Fig. 3e; Supplementary Fig. 6). The number of CD4^+^ lymphocytes did not differ between nintedanib-treated and vehicle-treated tumours (Supplementary Fig. 7). Together, these observations indicated that activated CD8^+^ T cells contribute to the antitumour efficacy of nintedanib.

### Nintedanib inhibits the proliferation and activation of fibroblasts

**Fig. 4:**
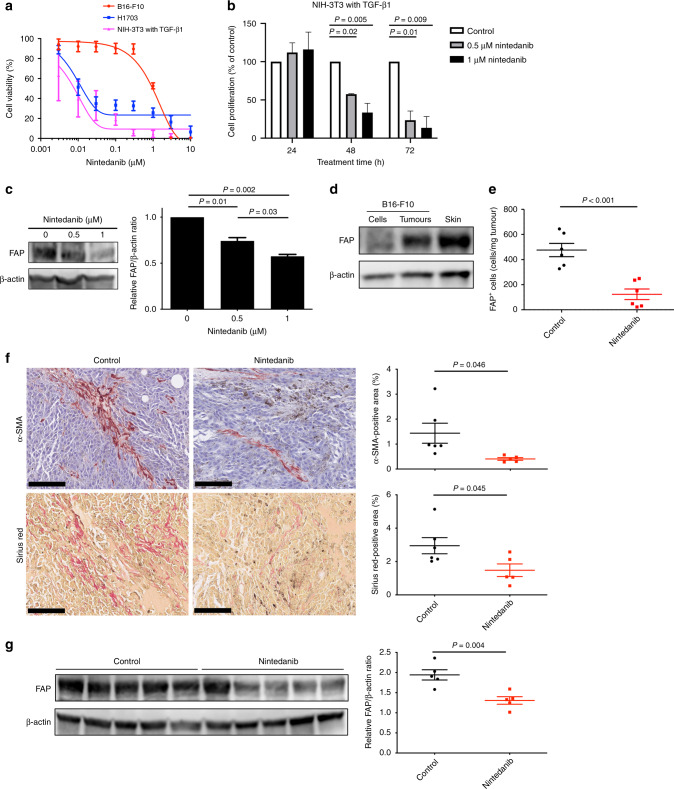
Nintedanib inhibits both the proliferation and activation of fibroblasts. **a** Cell viability assay for nintedanib and either NIH-3T3 cells treated with TGF-β1, H1703 cells (positive control) or B16-F10 cells (negative control). Data are means ± SEM for two independent experiments, each performed with six technical replicates. **b** Cell proliferation assay for nintedanib and TGF-β1–stimulated NIH-3T3 cells. The cells were treated with 0.5 or 1 μM nintedanib for 24, 48 or 72 h, after which living cells were counted by flow cytometry and normalised by those in untreated samples. Data are means + SEM for two independent experiments, each performed with two technical replicates. *P* values were determined by one-way ANOVA with Tukey’s correction for multiple comparisons and are shown only if <0.05. **c** Immunoblot analysis of FAP and β-actin (loading control) (left) as well as densitometric quantification of the FAP/β-actin ratio (right) for NIH-3T3 cells stimulated with TGF-β1 and exposed to the indicated concentrations of nintedanib. The quantitative data are means + SEM for two independent experiments. *P* values were determined by one-way ANOVA with Tukey’s correction for multiple comparisons. **d** Immunoblot analysis of FAP and β-actin in lysates of cultured B16-F10 cells, B16-F10 tumour tissue, and normal mouse skin tissue (positive control). **e** Flow cytometric analysis of FAP^+^ cells in B16-F10 tumours derived from mice treated with vehicle or nintedanib for 7 days. Data are expressed as number of FAP^+^ cells per milligram of wet tumour weight and are means ± SEM for six mice per group. The *P* value was determined with the unpaired *t*-test. **f** Representative immunohistochemical staining of α-SMA and Sirius red staining of collagen (left) as well as the corresponding percentage positive areas (right) for B16-F10 tumours derived from mice treated with vehicle or nintedanib for 7 days. Scale bars, 100 μm. The quantitative data are means ± SEM for five or six mice per group. The *P* values were determined with the unpaired *t*-test. **g** Immunoblot analysis of FAP and β-actin (left) as well as densitometric quantification of the FAP/β-actin ratio for lysates of B16-F10 tumour tissue derived from mice treated with vehicle or nintedanib for 7 days. The quantitative data are means ± SEM for five mice per group. The *P* value was determined with the unpaired *t-*test.

CAFs are the most abundant cell population in the TME and restrict the infiltration of CD8^+^ cells into tumour tissue, and nintedanib possesses antifibrotic activity.^4,12,14,24^ These previous findings and our results showing that nintedanib increased the number of CD8^+^ TILs in B16-F10 tumours suggested that nintedanib might promote the antitumour immune response by targeting CAFs. To explore this hypothesis, we first evaluated the antifibrotic activity of nintedanib with NIH-3T3 mouse fibroblast cells. Immunoblot analysis revealed that these cells express both PDGFR-α and FGFR-2 but not VEGFR-2 (Fig. 1a; Supplementary Fig. 8), suggesting that nintedanib is a potential targeted agent for these cells. We found that nintedanib attenuated the viability and proliferation of NIH-3T3 cells stimulated with TGF-β1 (Fig. 4a, b), a profibrotic cytokine that activates fibroblasts by triggering their conversion to myofibroblasts.^15^ We further explored whether nintedanib might affect the activation of fibroblasts in vitro by assessing the expression of fibroblast activation protein (FAP), which is selectively expressed at a high level on myofibroblasts.^25^ Immunoblot analysis revealed that treatment with nintedanib induced a concentration-dependent reduction in FAP expression in TGF-β1–stimulated NIH-3T3 cells (Fig. 4c). Our in vitro results thus showed that nintedanib inhibits both the proliferation and activation of fibroblasts.

To verify the antifibrotic effects of nintedanib in vivo, we first evaluated whether FAP could be detected in the B16-F10 tumour stroma. Immunoblot analysis detected FAP in lysates of B16-F10 tumours but not in those of cultured B16-F10 cells (Fig. 4d), suggesting that FAP-expressing CAFs are present in tumour stromal tissue. Flow cytometric analysis also revealed the presence of FAP in B16-F10 tumour tissue resected from mice, but not in B16-F10 cells cultured in vitro (Supplementary Fig. 9), supporting the notion that the B16-F10 tumour stroma contains FAP^+^ CAFs. Moreover, an ELISA showed that FGF-2, which is secreted by CAFs,^12^ was present in B16-F10 tumour lysates but not in lysates or conditioned medium of B16-F10 cells cultured in vitro (Supplementary Fig. 10), suggesting that CAFs in the tumour stroma are functional in terms of FGF-2 production. We explored whether nintedanib might affect the number of FAP^+^ CAFs in B16-F10 tumours by performing flow cytometric analysis. Such analysis revealed that the number of FAP^+^ CAFs was significantly reduced in tumours from nintedanib-treated mice compared with those from control mice (Fig. 4e). Immunohistochemical analysis also showed that nintedanib treatment reduced the number of α-SMA^+^ CAFs as well as the extent of collagen deposition in B16-F10 tumours (Fig. 4f; Supplementary Fig. 11). Furthermore, immunoblot analysis revealed that FAP expression was significantly reduced in tumours from nintedanib-treated mice compared with those from control mice (Fig. 4g). Together, these findings indicated that FAP^+^ CAFs that express FGF-2 are present in the B16-F10 tumour stroma and that nintedanib efficiently blocks the proliferation and activation of these cells, with these effects possibly leading to attenuation of the immunosuppression exerted by the TME and an increase in the infiltration and activation of CD8^+^ TILs.

### Increased antitumour efficacy of combined treatment with nintedanib and PD-1 blockade in vivo

**Fig. 5:**
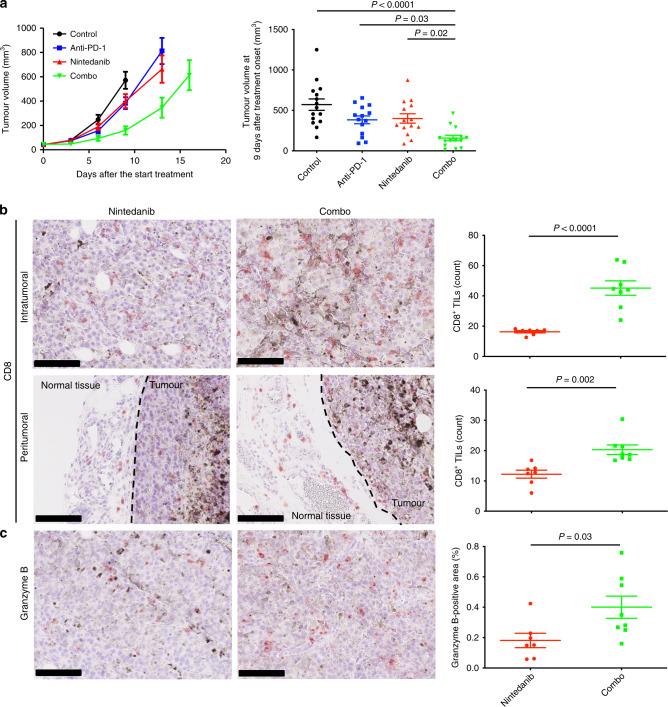
The combination of nintedanib and PD-1 blockade shows an increased antitumour efficacy. **a** Time course of tumour volume (left) as well as tumour volume at 9 days after treatment initiation (right) for subcutaneous B16-F10 tumours treated with anti–PD-1, nintedanib or the combination of both agents. Data are means ± SEM for 14 or 15 mice in each arm (pooled from two independent experiments). *P* values were determined by one-way ANOVA with Tukey’s correction for multiple comparisons and are shown only if <0.05. **b** Representative immunohistochemical staining of CD8 (left) as well as the number of CD8^+^ TILs (right) for intra- and peritumoural regions of B16-F10 tumours derived from mice treated with nintedanib or the combination of anti–PD-1 and nintedanib for 7 days. Scale bars, 100 μm. **c** Representative immunohistochemical staining (left) as well as the percentage positive area (right) for granzyme B in B16-F10 tumours derived from mice treated with nintedanib or the combination of anti–PD-1 and nintedanib for 7 days. Scale bars, 100 μm. Quantitative data in **b** and **c** are means ± SEM for seven or eight mice per group. *P* values in **b** and **c** were determined with the unpaired *t*-test.

Anticancer agents that target immunosuppression in the TME have recently been shown to promote the antitumour immune response and to enhance the efficacy of immunotherapy.^5,26^ We therefore hypothesised that nintedanib might be a rational partner for ICB therapy. To address this possibility, we investigated the antitumour efficacy of the combination of nintedanib with an antibody to PD-1 in the B16-F10 syngeneic mouse model. The combination treatment resulted in a significant delay in tumour growth compared with either nintedanib or PD-1 blockade alone (Fig. 5a). No pronounced toxicity such as loss of body weight was observed for mice in any of the four treatment groups. Immunohistochemical analysis showed that the combination treatment increased both the number of CD8^+^ TILs and granzyme B production in B16-F10 tumour tissue compared with treatment with nintedanib alone (Fig. 5b, c). Overall, these results suggested that nintedanib increased the sensitivity of tumours to PD-1 blockade therapy by promoting immune activation.

## Discussion

Our in vitro and in vivo results have suggested that nintedanib exerts indirect antitumour activity in B16-F10 tumour-bearing mice by targeting the TME including immune cells and fibroblasts. This activity thus appeared to be mediated by promotion of the infiltration and activation of CD8^+^ TILs and suppression of the proliferation and activation of CAFs. Nintedanib also showed enhanced antitumour efficacy in combination with PD-1 blockade.

Growth factors such as platelet-derived growth factor (PDGF), FGF and VEGF are important drivers of fibrosis, with this effect being mediated by binding of these factors to their corresponding receptors on fibroblasts and consequent stimulation of the proliferation of these cells.^12,27,28^ Nintedanib binds competitively to the ATP binding pocket of these receptors and thereby inhibits the ligand-induced proliferation of lung fibroblasts.^12–14^ This antiproliferative effect of nintedanib is not limited to the lung, however, with this agent also having been shown to attenuate the PDGF-induced proliferation and migration of dermal fibroblasts derived from individuals with systemic sclerosis.^24^ In the clinical setting, treatment with nintedanib has been found to slow disease progression in patients with idiopathic pulmonary fibrosis by attenuating the loss of lung function.^11^ On the basis of the experimental results showing that it inhibits fundamental processes of fibrosis regardless of the organ and its established antifibrotic clinical efficacy in patients with fibrosis-related disease, nintedanib has been investigated as a potential targeted agent for CAFs. A preclinical study thus showed that nintedanib inhibited both the proliferation and activation of patient-derived lung CAFs in vitro,^15^ a finding consistent with our present data.

Various therapeutic strategies have been developed for FAP-expressing CAFs or their functional mediators. Genetic ablation or pharmacological inhibition of FAP resulted in a reduction in the extent of myofibroblast infiltration and was associated with slower tumour growth in preclinical models.^29^ FAP^+^ CAFs were also shown to induce immunosuppression by excluding CD8^+^ T cells from the TME in a manner dependent on CXCL12-CXCR4 signalling; inhibitors of the chemokine receptor CXCR4 thus induced T cell accumulation and acted synergistically with an inhibitor of the PD-1 ligand PD-L1 in a mouse model of pancreatic cancer.^30^ Moreover, dipeptidyl peptidase 4 (DPP4) expressed on CAFs was shown to dimerise with FAP and thereby to interact with regulatory T cells to suppress the immune response.^31,32^ Blockade of DPP4 with the anti-diabetic drug sitagliptin increased the number of effector T cells and reduced tumour growth in mice.^31,33^ These previous studies have demonstrated that FAP^+^ CAFs confer an immunosuppressive environment and that elimination of these cells promotes the antitumour immune response. They therefore support our suggestion that targeting of FAP^+^ CAFs with nintedanib might alleviate CAF-mediated immunosuppression in the TME and thereby promote the accumulation of CD8^+^ TILs.

A Phase 1b/2 clinical trial of patients with advanced non–small cell lung cancer, including some who had been previously treated with ICB therapy, recently showed that the combination of nintedanib with the PD-1 inhibitor nivolumab and the CTLA-4 inhibitor ipilimumab was well tolerated and conferred clinical benefit, with 2 (17%) of 12 patients achieving a partial response (ClinicalTrials.gov identifier NCT03377023).^34^ One (14%) of seven patients who experienced disease progression after prior ICB therapy also achieved a partial response to the combination treatment,^34^ suggesting that this regimen has the potential to overcome ICB resistance. In addition, a basket Phase 1 study of patients with advanced solid tumours found that the combination of nintedanib and the PD-1 inhibitor pembrolizumab exerted a substantial antitumour effect, with a response rate of 25% (3 of 12 patients) (NCT02856425).^35^ In a cohort of patients with malignant pleural mesothelioma, 6 (21%) individuals achieved a partial response to this regimen.^36^ Our preclinical data show that the combination of PD-1 blockade and nintedanib as a CAF-targeted agent had an enhanced antitumour effect, supporting the rationale for these ongoing trials.

In conclusion, our findings indicate that nintedanib promotes antitumour immunity by targeting CAFs, and that the combination of nintedanib and PD-1 blockade shows enhanced antitumour efficacy. Clinical evaluation of nintedanib as a CAF-targeted agent in combination with antibodies to PD-1 is thus warranted.

## Supplementary information

Supplementary Figures

## Data Availability

The datasets generated and analysed in the study are available from the corresponding author on reasonable request.
