# Supplementary material for: Nintedanib promotes antitumour immunity and shows antitumour activity in combination with PD-1 blockade in mice: potential role of cancer-associated fibroblasts
Source: Br J Cancer. 2020 Dec 10;124(5):914–24. doi: 10.1038/s41416-020-01201-z (PMC7921555; doi:10.1038/s41416-020-01201-z)
Supplement: Supplementary file 1 — Supplementary Figures [file 41416_2020_1201_MOESM1_ESM.pdf]

Supplementary Figure 1

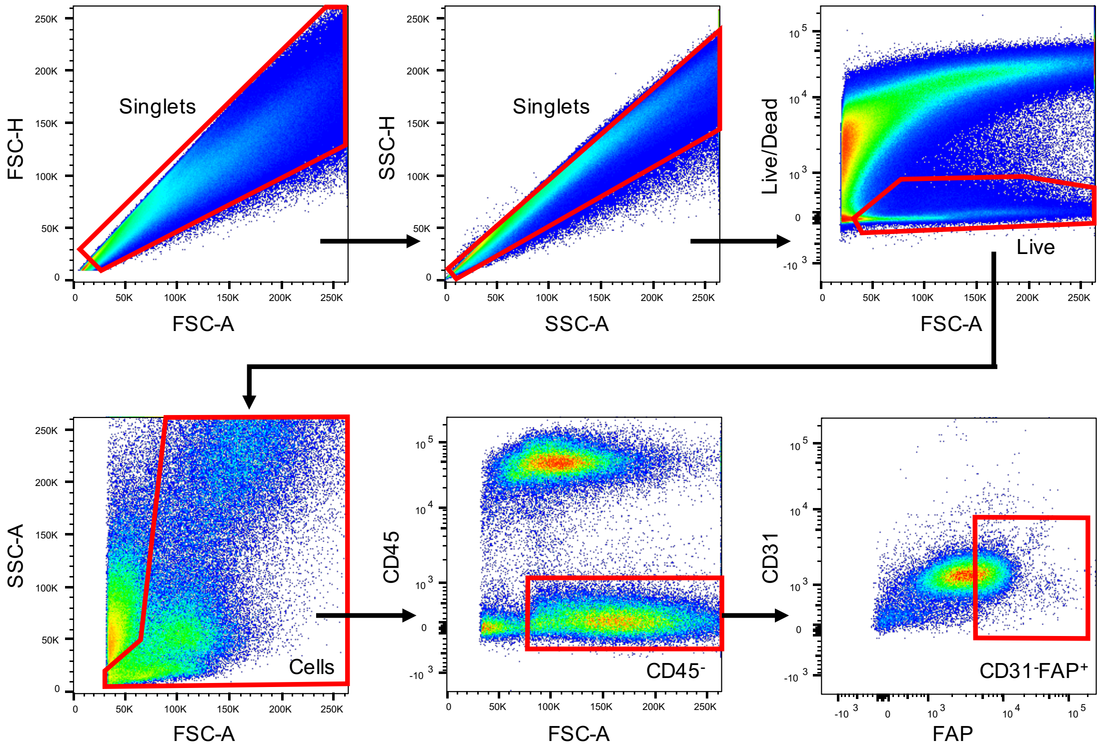

Supplementary Fig. 1. Gating strategy for flow cytometric analysis of FAP+ CAFs. Representative flow cytometric plots for B16-F10 tumors derived from mice with antibodies to FAP. FSC, forward scatter; SSC, side scatter.

Supplementary Figure 2

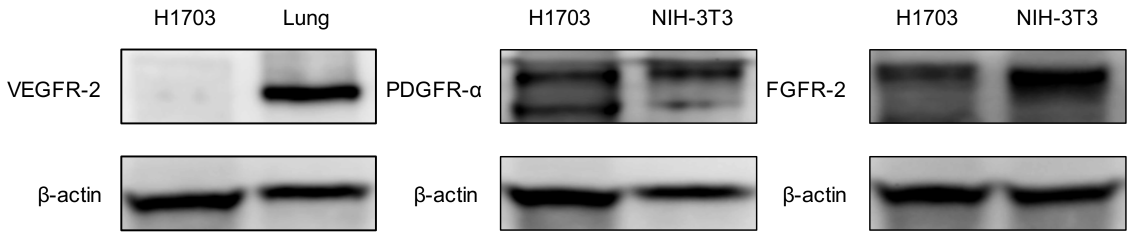

Supplementary Fig. 2. H1703 cells express PDGFR-α and FGFR-2. H1703 cells were subjected to immunoblot analysis of VEGFR-2, PDGFR-α, FGFR-2, and β-actin (loading control). Normal mouse lung tissue served as a positive control for VEGFR-2 expression, as did NIH-3T3 cells for PDGFR-α and FGFR-2 expression.

Supplementary Figure 3

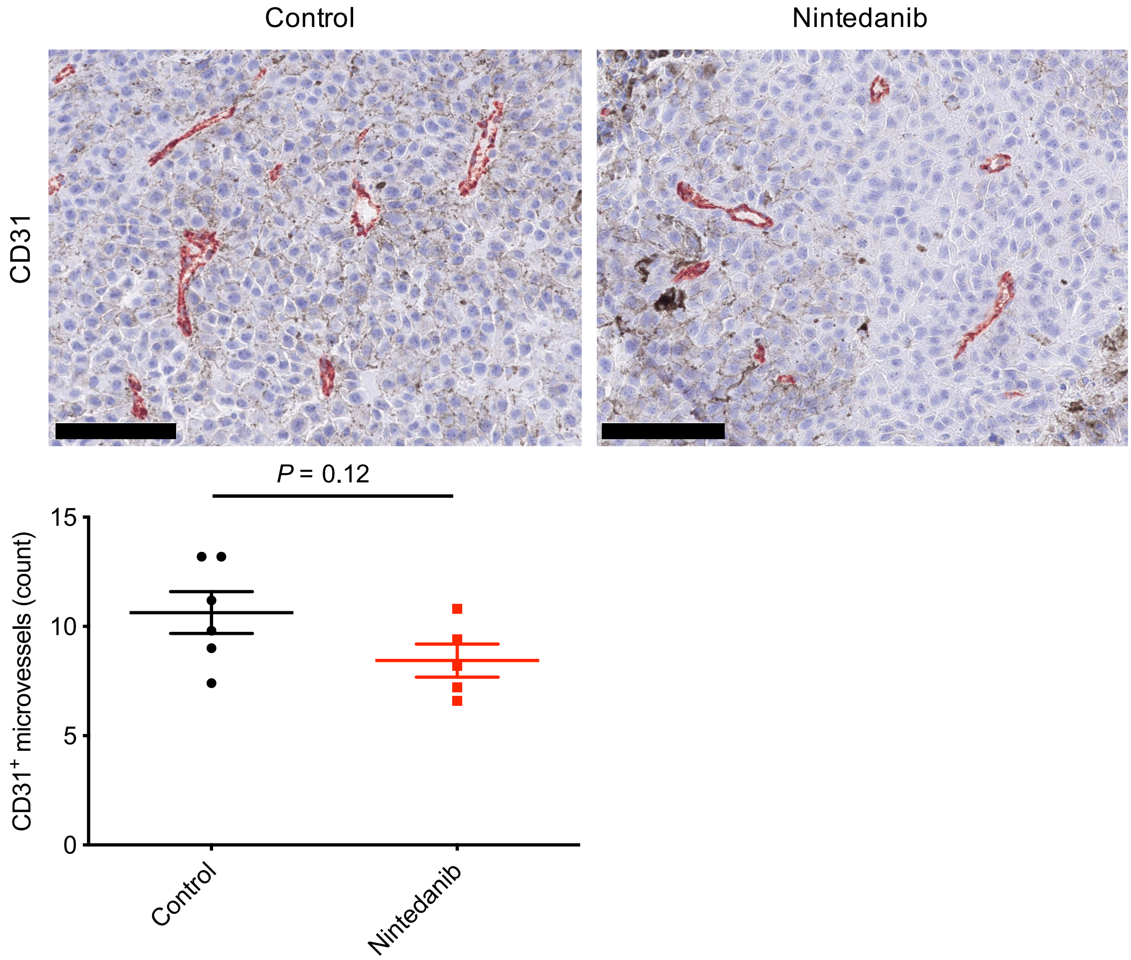

Supplementary Fig. 3. Nintedanib did not influence the number of CD31^{+} microvessel structures in B16-F10 tumors. Representative immunohistochemical staining for CD31 (top) and the number of CD31^{+} microvessel structures (bottom) are shown for B16-F10 tumors derived from mice treated with vehicle or nintedanib for 7 days. Scale bars, 100 µm. Quantitative data are means ± SEM for five or six mice per group. The *P* value was determined with the unpaired *t* test.

Supplementary Figure 4

Rank	Biological process	*P* value	ID
6	Response to interferon-beta	2.1E-13	GO:0035456
7	Response to organic substance	8.6E-12	GO:0010033
8	Innate immune response	1.4E-11	GO:0045087
9	Cellular response to organic substance	4.1E-10	GO:0071310
10	Response to external stimulus	5.5E-9	GO:0009605
11	Cellular response to chemical stimulus	6.3E-9	GO:0070887
12	Response to biotic stimulus	8.1E-9	GO:0009607
13	Response to other organism	2.0E-8	GO:0051707
14	Response to external biotic stimulus	2.1E-8	GO:0043207
15	Response to interferon-gamma	3.4E-8	GO:0034341
16	Regulation of immune response	9.0E-8	GO:0050776
17	Regulation of immune system process	1.6E-7	GO:0002682
18	Immune effector process	2.7E-7	GO:0002252
19	Response to bacterium	3.3E-7	GO:0009617
20	Cellular response to interferon-gamma	2.0E-6	GO:0071346

Supplementary Fig. 4. Gene ontology analysis reveals that nintedanib influences antitumor immunity. Up-regulated genes (>1.5-fold change in expression) as determined by microarray analysis in nintedanib-treated versus vehicle-treated B16-F10 tumors (*n* = 4 for each) were subjected to GO analysis by DAVID. Significantly enriched biological processes ranked 6 to 20 are listed. The *P* values were determined by Fisher's exact test, with the Bonferroni correction being applied for multiple comparisons.

Supplementary Figure 5

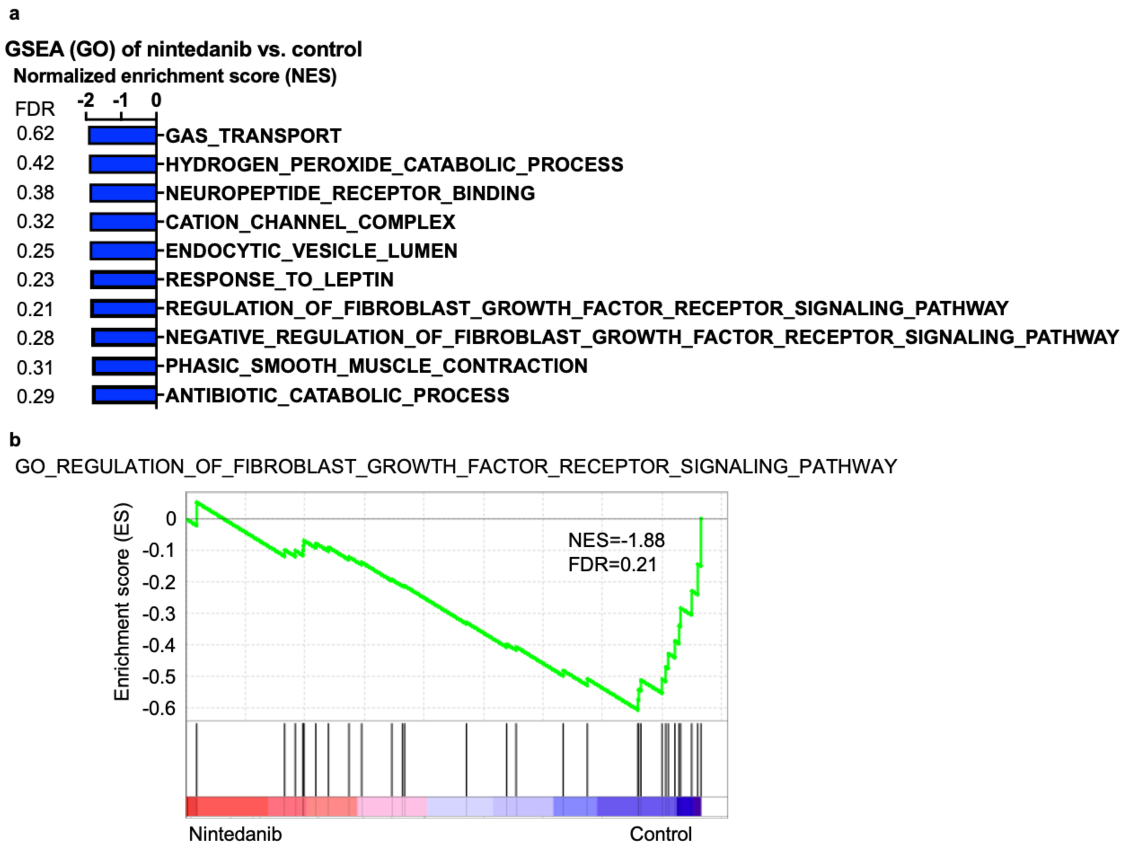

Supplementary Fig. 5. Gene signatures associated with fibroblasts were down-regulated in nintedanib-treated tumors. a GSEA of down-regulated gene signatures in nintedanib-treated versus control tumors (*n* = 4 for each) as determined by microarray analysis. The top 10 gene signatures from the Gene Ontology (GO) collection of MSigDB are listed. FDR, false discovery rate. **b** GSEA plot of enrichment for the gene signature related to regulation of FGFR signaing pathway from MSigDB for nintedanib-treated versus control tumors.

Supplementary Figure 6

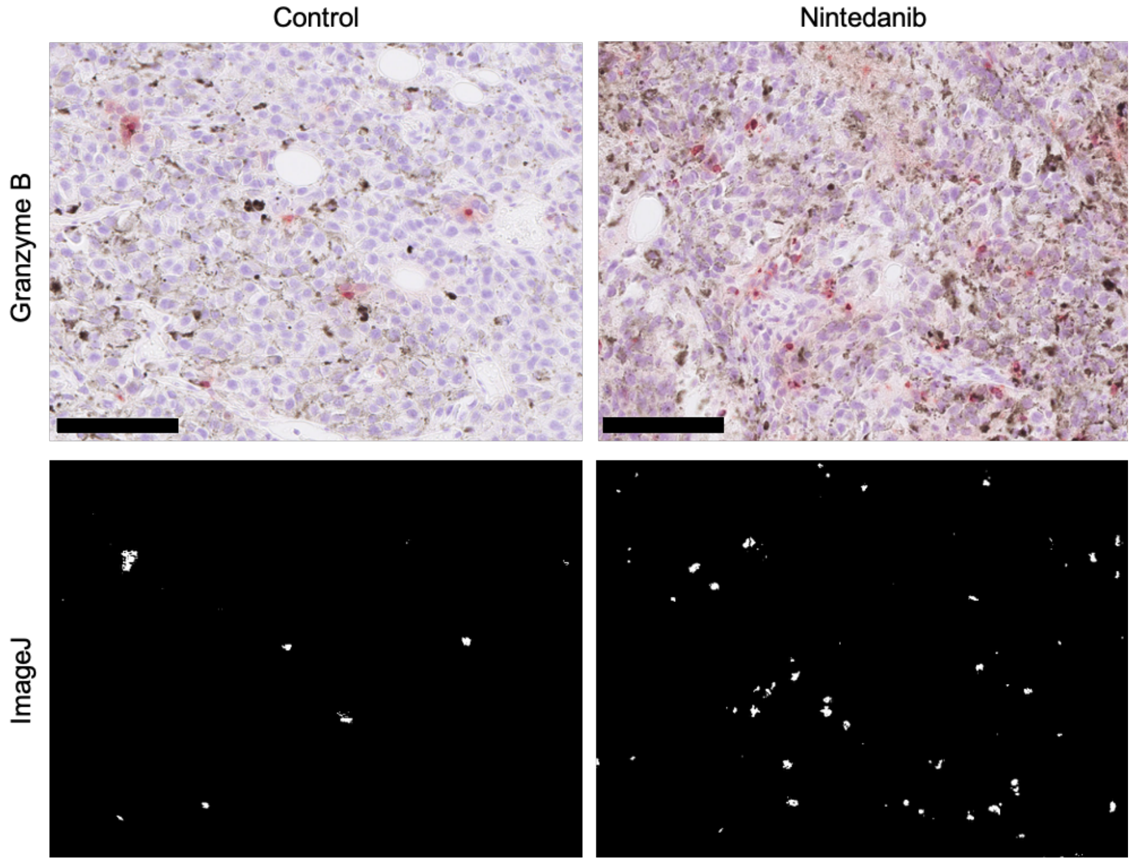

Supplementary Fig. 6. Quantitation of the positive area for granzyme B with Image J software. Representative immunohistochemical staining for granzyme B in B16-F10 tumors derived from mice treated with vehicle or nintedanib for 7 days is shown together with corresponding images analyzed with Image J software. The white areas in the latter images represent expression of granzyme B. Scale bars, 100 μm.

Supplementary Figure 7

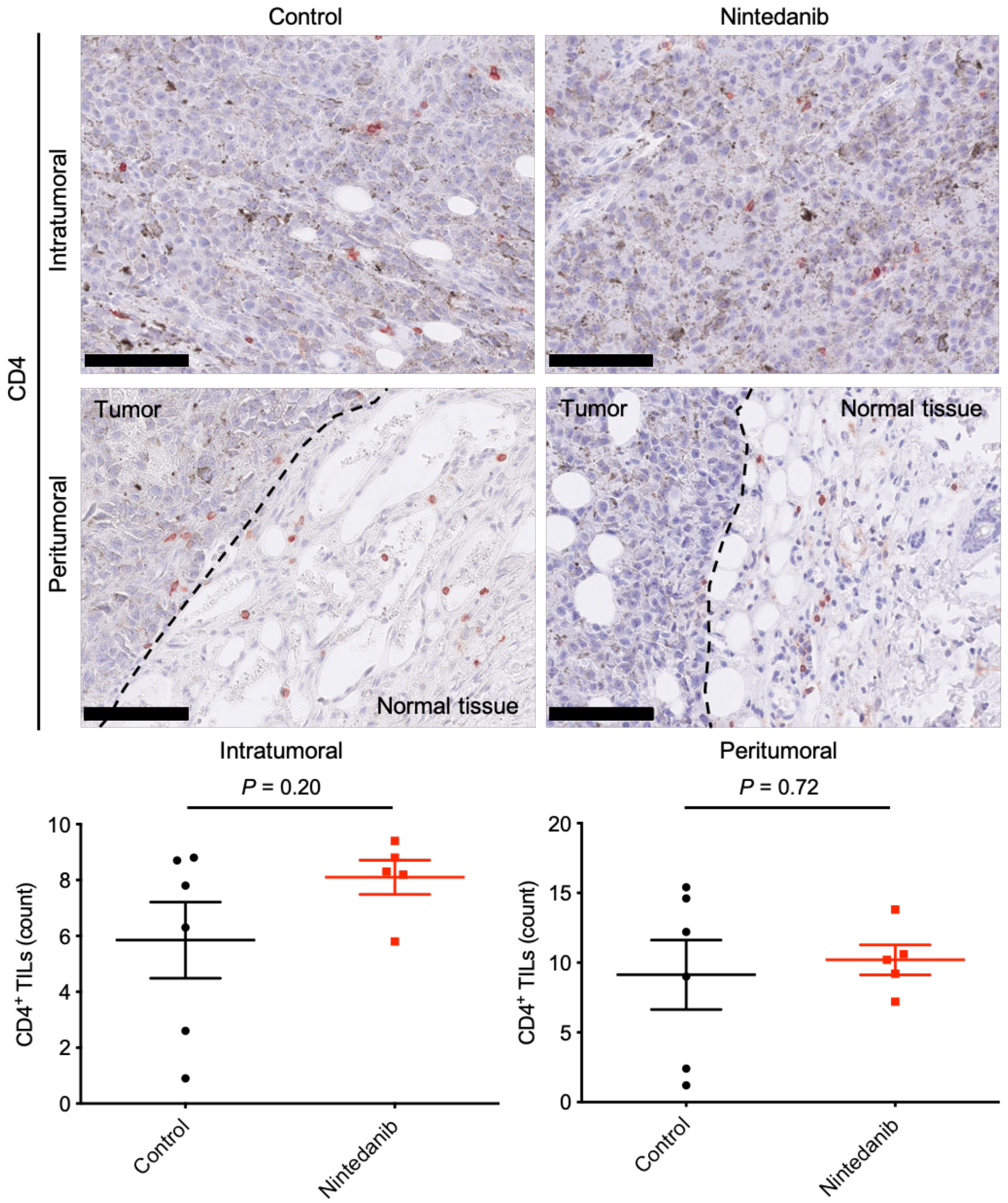

Supplementary Fig. 7. The number of CD4+ lymphocytes did not differ between nintedanib-treated and vehicle-treated tumors. Representative immunohistochemical staining for CD4 (top) and the number of CD4+ TILs (bottom) are shown for B16-F10 tumors derived from mice treated with vehicle or nintedanib for 7 days. Scale bars, 100 µm. Quantitative data are means ± SEM for five or six mice per group. The *P* values were determined with the unpaired *t* test.

Supplementary Figure 8

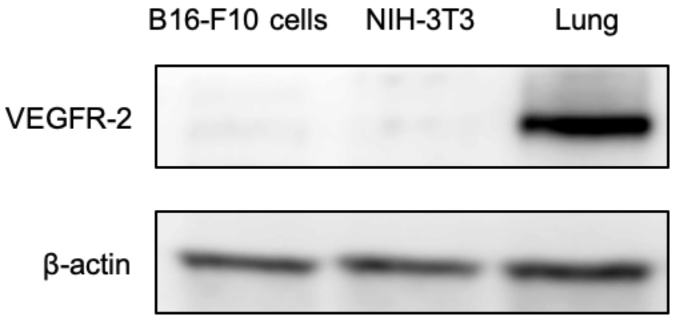

Supplementary Fig. 8. NIH-3T3 cells do not express VEGFR-2. NIH-3T3 and B16-F10 cells were subjected to immunoblot analysis of VEGFR-2 and β-actin (loading control). Normal mouse lung tissue served as a positive control for VEGFR-2 expression.

Supplementary Figure 9

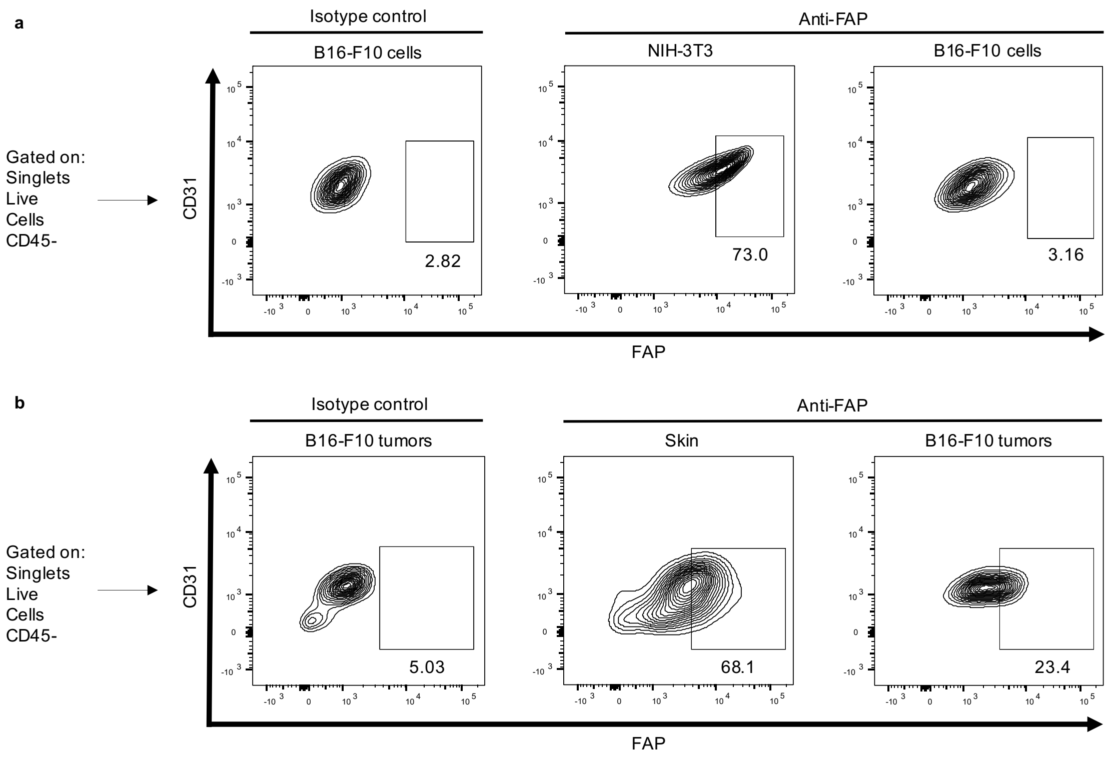

Supplementary Fig. 9. Flow cytometric analysis detected FAP expression in B16-F10 tumor tissue but not in cultured B16-F10 cells. a Representative flow cytometric plots for cultured B16-F10 cells with antibodies to FAP or isotype control antibodies (negative control) or for cultured NIH-3T3 cells with antibodies to FAP (positive control). The frequency of FAP-expressing cells is indicated. **b** Representative flow cytometric plots for B16-F10 tumor tissue with antibodies to FAP or isotype control antibodies (negative control) or for normal mouse skin tissue with antibodies to FAP (positive control). The frequency of FAP-expressing cells is indicated.

Supplementary Figure 10

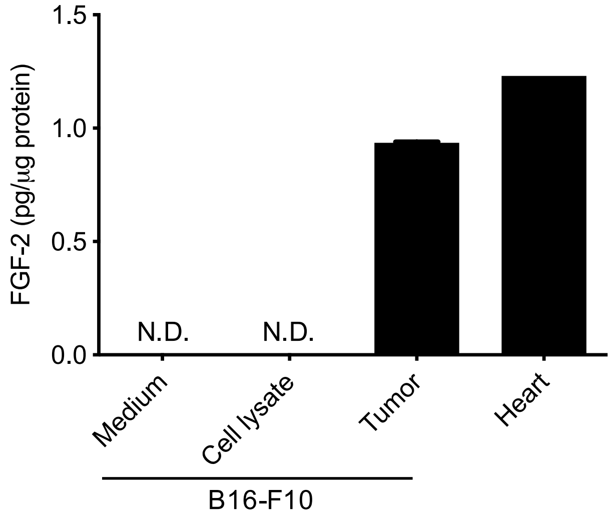

Supplementary Fig. 10. FGF-2 was present in B16-F10 tumor lysates but not in lysates or conditioned medium of cultured B16-F10 cells. FGF-2 content of cell lysates and conditioned medium for cultured B16-F10 cells as well as of lysates of B16-F10 tumor tissue or normal mouse heart tissue (positive control) as determined with an ELISA. Data are means of two technical replicates from one experiment. N.D., not detected.

Supplementary Figure 11

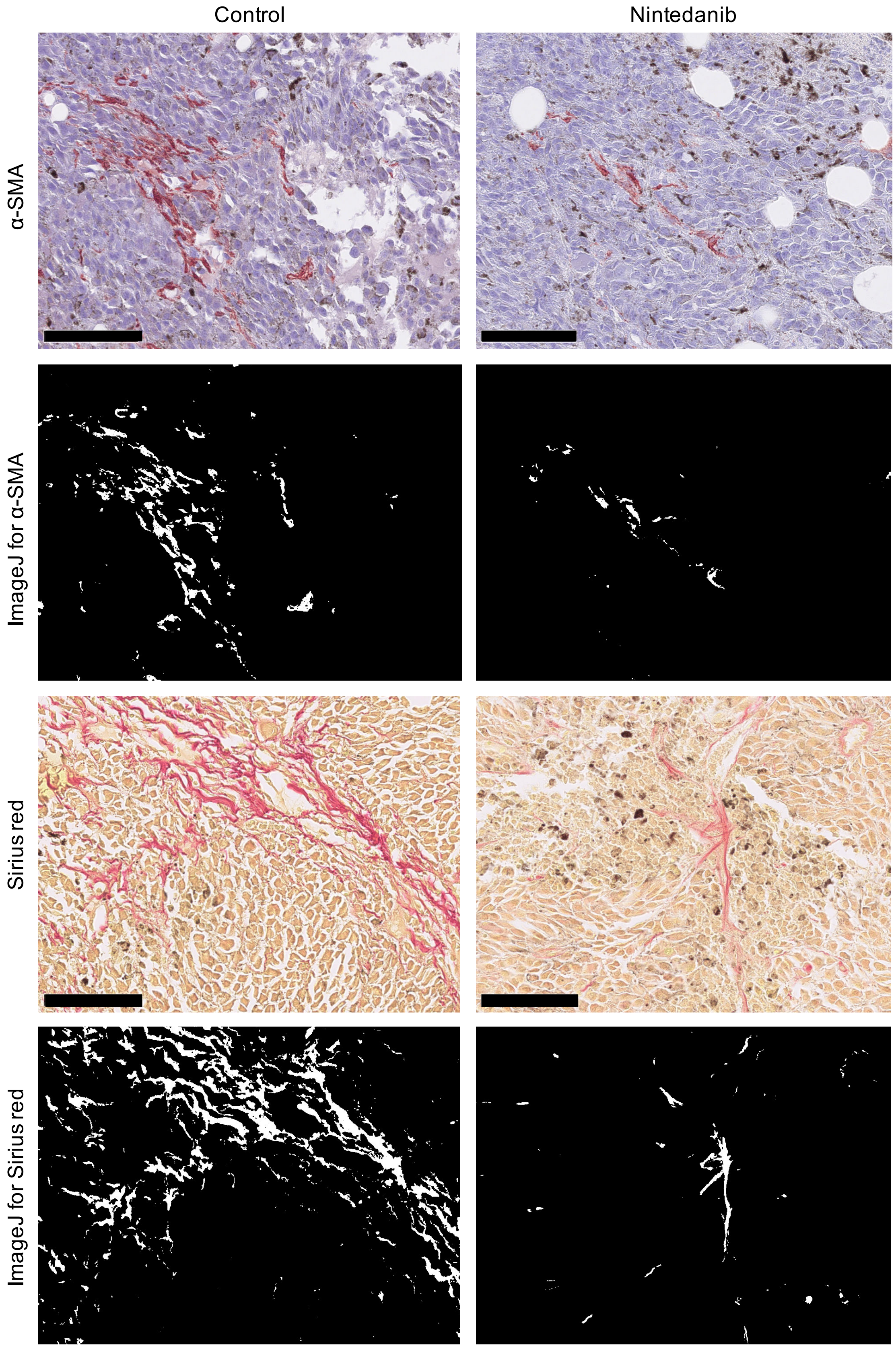

Supplementary Fig. 11. Quantification of the positive area for α-SMA and collagen with Image J software. Representative immunohistochemical staining for α-SMA and Sirius red staining for collagen in B16-F10 tumors derived from mice treated with vehicle or nintedanib for 7 days are shown together with corresponding images analyzed with Image J software. The white areas in the latter images represent expression of α-SMA and deposition of collagen, respectively. Scale bars, 100 μm.
